# Who Needs Endoscopic Removal of Food Residue From the Esophagus Before Peroral Endoscopic Myotomy for Esophageal Achalasia and Esophagogastric Junction Outflow Obstruction?

**DOI:** 10.1002/deo2.70196

**Published:** 2025-09-03

**Authors:** Masatoshi Kaizuka, Tetsuya Tatsuta, Yohei Sawada, Taka Asari, Shiro Hayamizu, Shinji Oota, Keisuke Hasui, Hidezumi Kikuchi, Hiroto Hiraga, Daisuke Chinda, Tatsuya Mikami, Shinsaku Fukuda, Hirotake Sakuraba

**Affiliations:** ^1^ Department of Gastroenterology Hematology and Clinical Immunology Hirosaki University Graduate School of Medicine Aomori Japan; ^2^ Department of Community Medicine Hirosaki University Graduate School of Medicine Aomori Japan; ^3^ Division of Endoscopy Hirosaki University Hospital Aomori Japan; ^4^ Department of Preemptive Medicine Hirosaki University Graduate School of Medicine Aomori Japan

**Keywords:** anesthesia, endoscopy, esophageal achalasia, esophageal motility disorders, esophagus

## Abstract

**Objectives:**

Peroral endoscopic myotomy (POEM) is an established treatment for esophageal achalasia. Despite favorable outcomes, the risk of reflux aspiration during general anesthesia induction in POEM remains a concern. This study aimed to identify the risk factors for food residue in the esophagus before POEM and evaluate the necessity of esophagogastroduodenoscopy (EGD) and cleansing the day before POEM.

**Methods:**

A retrospective analysis of 61 patients with esophageal achalasia and esophagogastric junction outflow obstruction undergoing POEM between July 2017 and March 2024 was performed. Patients were divided into two groups based on the presence of food residue observed during preoperative EGD: residual (*n* = 16) and no‐residual (*n* = 45) food groups. The factors compared included age, sex, Chicago criteria, duration of symptoms, Eckardt score, integrated relaxation pressure, esophageal dilation grade and type on esophagography, and presence of residual food during the initial EGD.

**Results:**

In univariate analysis, residual food was more common in patients aged <60 years (*p* < 0.05) and those with higher esophageal dilation grades (*p* < 0.05). Additionally, residual food during the initial EGD was identified as a significant predictor (*p* < 0.05).

**Conclusions:**

Preoperative EGD and esophageal cleansing the day before POEM may be warranted in patients with initial EGD‐detected residue, younger age, and marked dilation, to reduce reflux aspiration risk and improve procedural safety.

## 1 Introduction

Peroral endoscopic myotomy (POEM) is performed for esophageal motility disorders (EMDs), including esophageal achalasia, which is characterized by incomplete lower esophageal sphincter (LES) relaxation and abnormal esophageal contractions, and its outcomes are favorable [1]. In addition, this technique can reduce the physical invasiveness [2].

POEM is initiated under general anesthesia with positive pressure ventilation [3]. After local injection of saline into the submucosa of the esophagus, a 2‐cm longitudinal mucosal incision is made. A submucosal tunnel is created from the entry to the LES to approximately 2–3 cm on the gastric side. The inner circular muscle on the gastric side is incised. Finally, the entry is closed using clips.

Reflux aspiration occurs at a frequency of 3/10,000 cases with the induction of general anesthesia for various surgeries [4]. Patients with EMD, characterized by incomplete LES relaxation (esophageal achalasia and esophagogastric junction outflow obstruction [EGJOO]), tend to have more esophageal residue than those without EMD with incomplete LES relaxation. There have been several case reports of reflux aspiration during the induction of general anesthesia in patients with esophageal achalasia [5, 6]. However, no detailed studies have investigated the risk factors for reflux aspiration. The induction of general anesthesia in these patients is thought to carry a higher risk of reflux aspiration than in patients with normal LES relaxation.

One preventive measure against reflux aspiration during the induction of general anesthesia in patients undergoing POEM is to perform esophagogastroduodenoscopy (EGD) the day before and remove any food residue. According to a survey conducted among POEM facilities worldwide (in Asia, North America, and Europe), cleansing was performed the day before in six out of 16 facilities (37.5%) [7].

If food residue is observed during the initial EGD, which is often performed several months before POEM, it is necessary to perform EGD and cleanse the day before POEM. However, even if cleansing on the previous day is deemed unnecessary, occasional instances exist where a large amount of residue is observed at the beginning of the POEM procedure.

Currently, it remains unclear which patients require EGD and cleansing the day before POEM. However, we believe that identifying the factors related to the presence of residues will reduce reflux aspiration during anesthesia induction and allow for the safe performance of POEM.

Therefore, in this study, we clarified the risk factors for food residue in the esophagus and identified patients requiring endoscopic cleansing of the esophagus the day before POEM.

## 2 Methods

### 2.1 Study Design

This was a single‐center retrospective study, and its protocol was approved by the Ethics Committee of Hirosaki University (2023‐032).

### 2.2 Definition of Food Residue

**FIGURE 1:**
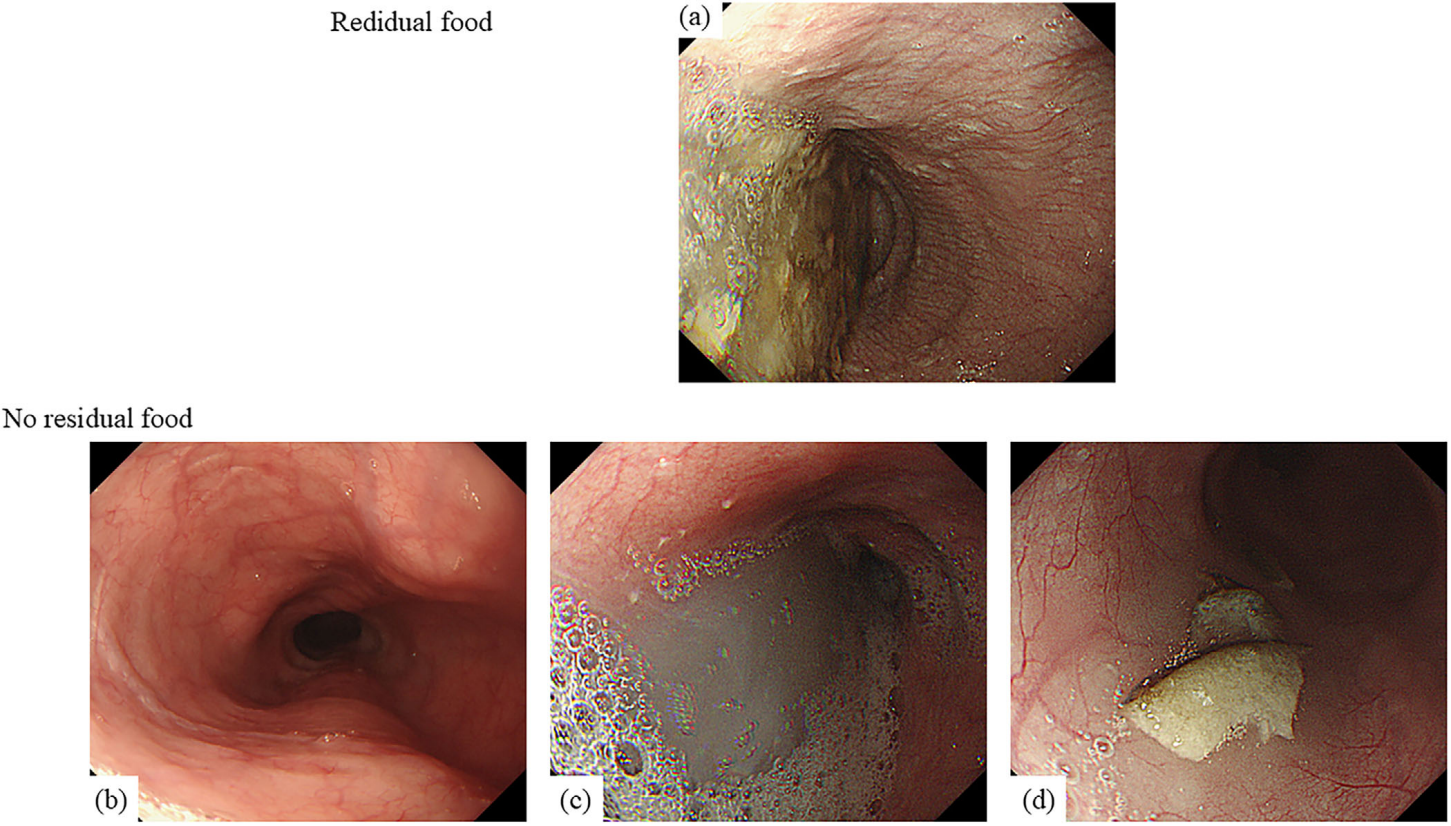
Endoscopic images of patients with esophageal achalasia: (a) A Large amount of food residue in the esophagus, and difficult to suction by endoscope. (b) No liquid retention or food residue in the esophagus. (c) Some liquid retention in the esophagus, yet no food residue. (d) A small amount of food residue is in the esophagus. Endoscopic suction is possible. In this study, we define (a) as “with food residue” and (b–d) as “without food residue”.

“Food residue” was defined as a large amount of residue observed immediately after inserting the endoscope into the esophagus (Figure 1a). When no residue was present (Figure 1b), only liquid was present (Figure 1c), or only a small amount of residue that can be easily suctioned was present (Figure 1d), we defined it as “without food residue.”

### 2.3 Patients

**FIGURE 2:**
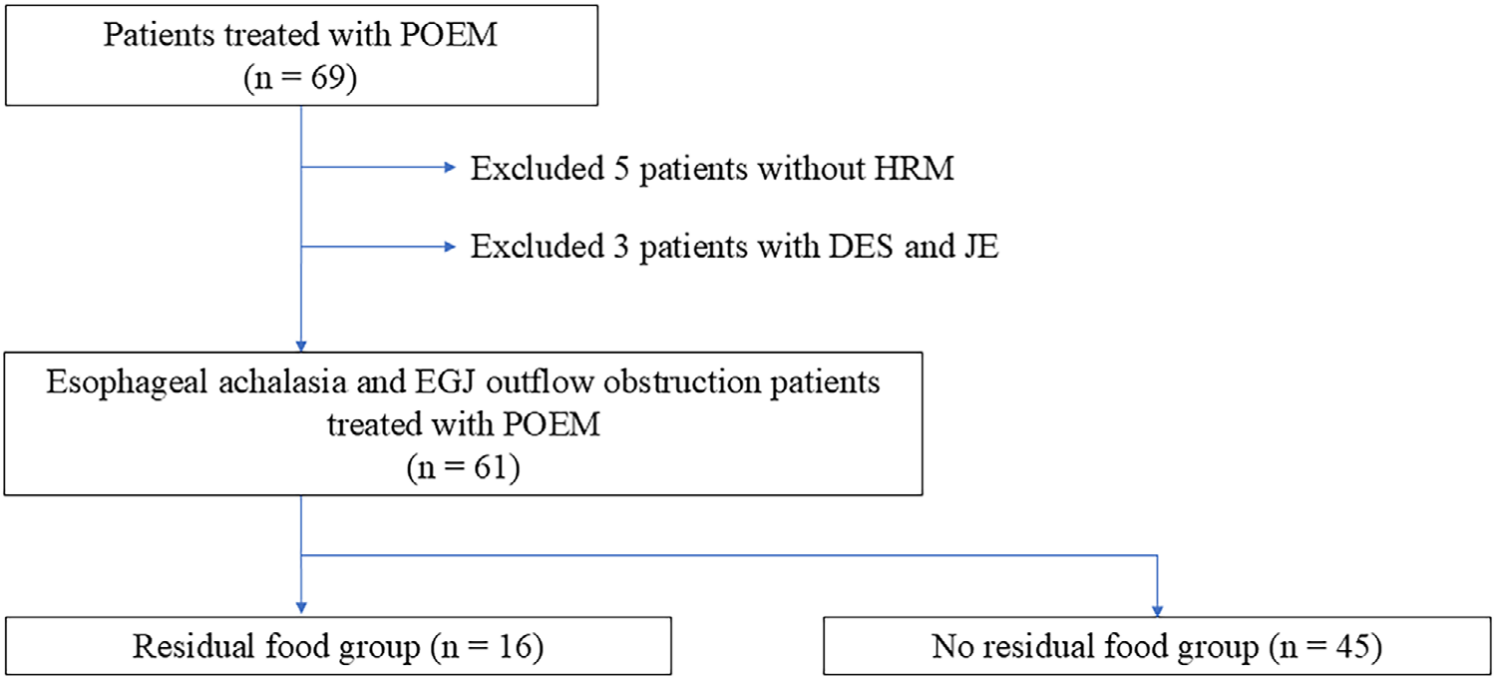
Flow chart for participant selection. Flowchart of participant selection in this study. POEM was performed on 69 patients between July 2017 and March 2024, POEM was performed in 69 cases. Among them, 61 cases were analyzed after excluding five cases without HRM and three cases with DES and JE. Sixteen patients were classified into the residual food group on residual food at the EGD day before POEM (*n* = 13) or on the start of POEM (*n* = 3). Additionally, 45 patients were classified into the no residual food group had no residual food on the EGD (the day before POEM, *n* = 21) and at the start of POEM (*n* = 24). DES, distal esophageal spasm; EGJOO, esophagogastric junction outflow obstruction; HRM, high‐resolution manometry; JE, Jackhammer esophagus; POEM, peroral endoscopic myotomy.

POEM was performed in 69 patients between July 2017 and March 2024 (Figure 2). Among these patients, 61 who underwent high‐resolution manometry (HRM) and were diagnosed with esophageal achalasia or EGJOO were included in the analysis. All patients fasted from the day before the POEM procedure, with only water permitted. The patients were intubated in the supine position with the head elevated to reduce reflux. Sixteen patients with residual food observed on EGD and difficulty in performing cleansing by endoscopy were classified into the residual food group. Among them, 13 had residual food observed on EGD the day before POEM, and three had residual food observed at the start of POEM. Additionally, 45 patients without residual food on EGD or with a small amount of residue that could be suctioned by endoscopy were classified into the no residual food group. Moreover, 21 patients had no residual food on EGD the day before POEM, and 24 had no residual food at the start of POEM. A flowchart of the study is shown in Figure 2. The time required to clean the residual food the day before or immediately before POEM was investigated in the residual food group.

The following variables were compared between both groups: age, sex, Chicago criteria, duration of symptom, Eckardt score, integrated relaxation pressure (IRP), esophageal dilation grade and type on esophagography, presence of prior treatment, presence of residual food at the initial EGD performed at our hospital several months prior to the POEM procedure (initial EGD), operation time, and C‐reactive protein (CRP) level on the first postoperative day (POD).

The same variables were compared between patients with and without reflux aspiration during anesthesia. Reflux aspiration was defined as the presence of residual material in the trachea or oral cavity, confirmed by post‐intubation bronchoscopy.

## 3 Examination of EMD

### 3.1 Eckardt Score

The Eckardt score is a symptom score for EMD that is the sum of the frequency of dysphagia, regurgitation, chest pain (0 points: no symptoms, 1 point: occasional symptoms, 2 points: daily symptoms, and 3 points: symptoms at every meal), and weight loss (0 points: none; 1 point: <5 kg; 2 points: 5–10 kg; and 3 points: >10 kg) [8]. The higher the score, the more pronounced the symptoms (maximum: 12), and the lower the score, the milder the symptoms (minimum: 0).

### 3.2 HRM Diagnosis

Each patient with EMD was diagnosed using esophagography, EGD, and HRM based on the Chicago Classification Criteria v3.0 [9]. In this study, we used the Starlet HRM system (StarMedical, Tokyo, Japan). The IRP was measured as the lowest 4‐s cumulative pressure value that occurred during a 10‐s post‐deglutition time window in the electronically generated e‐sleeve signal through the anatomic zone defining the esophagogastric junction [10]. IRP ≥ 26 mmHg was defined as LES relaxation failure on the Starlet HRM system [11]. Esophageal achalasia was diagnosed when the IRP was >26 mmHg and there was no normal peristalsis. Esophageal achalasia was further divided into three subtypes based on the type of esophageal contraction: Type I (100% failed peristalsis), II (≥20% panesophageal pressurization), and III (≥20% spastic contraction). EGJOO was diagnosed when the IRP was >26 mmHg with normal peristalsis.

The IRP was used as a parameter to evaluate esophagogastric junction relaxation [12]. HRM was measured as the mean value of the maximal relaxation of weakness over a 4‐s period beginning with upper esophageal sphincter relaxation with gastric pressure as the reference.

Several achalasia cases with normal IRP levels have been reported [13]. Esophageal achalasia with normal IRP was comprehensively diagnosed based on typical findings on esophagography (bird‐beak or sigmoid‐like appearance) and EGD (rosette‐like esophageal folds or pinstripe pattern) [14, 15].

### 3.3 Barium Esophagogram

The esophageal dilation grade was assessed based on the maximum transverse diameter (d) using a barium esophagogram [16], which was classified into: Grade I: d < 3.5 cm, Grade II: 3.5 cm ≤  d < 6.0 cm, and Grade III: d ≥ 6.0 cm. Esophageal type was classified by the angle of esophageal flection (α) using barium esophagography: St (straight type): α ≥ 135°, Sg (sigmoid type): 90° ≤ α < 135°, and aSg (advanced sigmoid type): α < 90°.

### 3.4 Statistical Analysis

Statistical analysis was performed using EZR [17]. Fisher's exact test was used to compare the ratios between groups. The Mann–Whitney U test was used to compare continuous variables between the two groups. Logistic regression analysis was used to identify predictive factors for esophageal food residue. Clinical variables showing a significant difference in the univariate analyses were included in the multivariate analysis. Odds ratios (ORs), sensitivities, and specificities were calculated. A receiver operating characteristic (ROC) curve was constructed and used to determine the cut‐off values detected at the point of maximum sensitivity and specificity. Statistical significance was set at *p* < 0.05.

## 4 Results

### 4.1 Patient Characteristics

Table 1 presents the clinical background characteristics of the residual and no‐residual food groups. The median (interquartile range [IQR]) age was 48 (42.0–56.3) and 61 (52.0–74.0) years in the residual and no‐residual food groups, respectively. Patients in the residual food group were significantly younger than those in the no‐residual food group (*p* < 0.05). The residual food group comprised 11 males and five females, whereas the no‐residual food group included 24 males and 21 females. Among the 16 patients in the residual food group, 15 had type I achalasia and one had type II. In the no‐residual food group (*n* = 45), 31 had Type I, nine had Type II, two had Type III achalasia, and three had EGJOO. Prior balloon dilation rates did not differ between groups (4 vs. 14; *p* = 0.757).

**TABLE 1:** Patient characteristics.

Characteristics	Residual food group (*n* = 16)	No residual food group (*n* = 45)	*p*‐Value
Age, years (median [IQR])	48.0 (42.0–56.3)	61.0 (52.0–74.0)	< 0.05
Sex, Male/Female	11/5	24/21	0.381
Diagnosis			0.333
Type I achalasia, *n* (%)	15 (93.8)	31 (68.9)	
Type II achalasia, *n* (%)	1 (6.2)	9 (20.0)	
Type III achalasia, *n* (%)	0 (0.0)	2 (4.4)	
EGJOO, *n* (%)	0 (0.0)	3 (6.7)	
Duration of symptom, years (median [IQR])	5 (3.5–11.0)	8 (4.0–16.0)	0.549
Eckardt score, points (median [IQR])	6 (4.5–7.0)	5 (4.0–7.0)	0.447
IRP, mmHg (median [IQR])	19.1 (13.7–22.6)	25.5 (15.5–31.5)	0.159
Dilation grade, Grade I/II/III	1/14/1	17/24/3	< 0.05
Type, St/Sg/aSg	8/5/3	16/21/7	0.507
Prior treatment			
Balloon dilation, *n* (%)	4 (25.0)	14 (31.1)	0.757
POEM, *n* (%)	0 (0.0)	0 (0.0)	
Surgery, *n* (%)	0 (0.0)	0 (0.0)	
Residual food at the initial EGD, *n* (%)	11 (68.8)	11 (24.4)	< 0.05
EGD the day before POEM, *n* (%)	13 (81.3)	21 (46.7)	< 0.05
POEM failure, *n* (%)	1 (6.2)	1 (2.2)	0.459
Operation time, min (median [IQR])	95 (77.0–98.5)	93 (77.5–105.5)	0.790
Cleansing time on the day before POEM, min (median [IQR])	2.5 (1–16)		
Cleansing time immediately before POEM, min (median [IQR])	4.5 (3.8–8.3)		
CRP on 1POD, mg/dL (median [IQR])	2.9 (2.1–4.2)	3.6 (2.1–5.3)	0.432

Grade I: d < 3.5 cm, Grade II: 3.5 cm ≤d <6.0 cm, Grade III: d ≧ 6.0 cm. St (Straight type): α ≥ 135°, Sg (Sigmoid type): 90° ≤α <135°, aSg (Advanced Sigmoid type): α < 90°. CRP, C‐reactive protein; EGD, esophagogastroduodenoscopy; EGJOO, esophagogastric junction outflow obstruction; IRP, integrated relaxation pressure; POD, post‐operative day.

### 4.2 Comparison Between the Residual and No‐Residual Groups

The median (IQR) duration of symptoms was 5 (3.5–11.0) and 8 (4.0–16.0) years in the residual and no‐residual food groups, respectively. The median (IQR) Eckardt score was 6 (4.5–7.0) and 5 (4.0–7.0) points in the residual and no‐residual food groups, respectively. The median (IQR) IRP was 19.1 (13.7–22.6) and 25.5 (15.5–31.5) mmHg in the residual and no‐residual food groups, respectively. The dilation grade was classified as grade I, II, and III in 1, 14, and 1 case, respectively, in the residual food group, and in 17, 24, and 3 cases, respectively, in the no‐residual group. The residual food group had a significantly higher percentage of grades II and III (*p* < 0.05) than the no‐residual food group. The esophageal types were St, Sg, and aSg in eight, five, and three patients, respectively, in the residual food group, and 16, 21, and three, respectively, in the no‐residual group. Thirteen patients (68.8%) had residual food at the initial EGD in the residual food group, and 11 (24.4%) had residual food in the no‐residual food group. The residual‐food group had a significantly higher percentage of residual food than the no‐residual food group at the initial EGD (*p* < 0.05). The median (IQR) operation time was 95 (77.0–98.5) and 93 (77.5–105.5) min in the residual and no‐residual food groups, respectively. Within the residual food group, the 13 patients who underwent EGD the day before required a median (IQR) of 2.5 (1–16) min for cleansing, while three who did not undergo EGD the day before required a median (IQR) of 4.5 (3.8–8.3) min for cleansing residual food immediately before POEM. The median (IQR) CRP levels on the first POD were 2.89 (2.06–4.17) and 3.63 (2.06–5.32) mg/dL in the residual and no‐residual food groups, respectively.

### 4.3 Risk Factor for Food Residue

Group comparisons in Table 2 were performed using Fisher's exact test. In the residual food group, the proportion of patients aged ≥60 years was lower, while the proportions of patients with esophageal dilation grades II and III, as well as those with residual food at the initial EGD, were significantly higher (*p* < 0.05).

**TABLE 2:** Comparison between the residual food group and the no residual food group.

	Residual food group (*n* = 16)	No residual food group (*n* = 45)	*p*‐Value
Age			
≥ 60 years, *n* (%)	2 (12.5)	24 (53.3)	< 0.05
Sex			
Male, *n* (%)	11 (68.8)	24 (53.3)	0.381
Duration of the symptom			
≥ 10 years, *n* (%)	5 (33.3)	19 (42.2)	0.762
Eckardt score			
≥ 6 points, *n* (%)	9 (60.0)	19 (42.2)	0.251
IRP			
≥ 26 mmHg, *n* (%)	3 (20.0)	21 (46.7)	0.079
Dilation grade			
Grade II and III, *n* (%)	15 (93.8)	27 (61.4)	< 0.05
Type			
Sigmoid, *n* (%)	8 (50.0)	28 (63.6)	0.383
Residual food at the initial EGD			
Yes, *n* (%)	11 (68.8)	11 (24.4)	< 0.05

EGD, esophagogastroduodenoscopy; IRP, integrated relaxation pressure.

In Table 3, univariate and multivariate analyses were conducted to compare the residual food group and the no‐residual food group. In the univariate analysis, patients in the residual food group were younger than those in the no‐residual food group (OR 0.13, 95% confidence interval [CI] 0.01–0.66, *p* < 0.05). In addition, the residual food group showed higher rates of grade II and III dilation (OR 9.18, 95% CI 1.08–419.67, *p* < 0.05) and residual food at the initial EGD (OR 6.55, 95% CI 1.66–29.84, *p* < 0.05) than the no‐residual food group. In the multivariate analysis, age < 60 years and residual food at the initial EGD were identified as independent predictive factors.

**TABLE 3:** Risk factors associated with residual food in patients with achalasia and esophagogastric junction outflow obstruction (EGJOO).

	Univariate		Multivariate	
	OR (95%CI)	*p*‐Value	OR (95%CI)	*p*‐Value
Age				
≥ 60 years	0.13 (0.01–0.66)	< 0.05	0.01 (0.01–0.41)	< 0.05
Sex				
Male	1.90 (0.51–8.18)	0.381	—	—
Duration of symptoms				
≥ 10 years	0.69 (0.16–2.67)	0.762	—	—
Eckardt score				
≥ 6 points	2.03 (0.54–7.87)	0.251	—	—
IRP				
≥ 26 mmHg	0.29 (0.05–1.29)	0.079	—	—
Dilation grade				
Grade II and III	9.18 (1.08–419.7)	< 0.05	5.68 (0.49–66.5)	0.166
Type				
Sigmoid	0.58 (0.15–2.14)	0.383	—	—
Residual food at the initial EGD				
Yes	6.55 (1.66–29.8)	< 0.05	14.7 (2.33–92.6)	< 0.05

CI, confidence interval; EGD, esophagogastroduodenoscopy; EGJOO, esophagogastric junction outflow obstruction; IRP, integrated relaxation pressure; OR, odds ratio.

**FIGURE 3:**
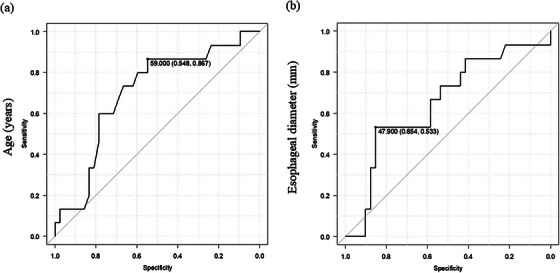
ROC curves. The ROC curve of age (a) and esophageal diameter (b) for the diagnosis of residual foods in the esophagus is shown. (a): The AUC is 0.702 (95% CI 0.548–0.867). The cutoff value of age is ≥ 59 years. The sensitivity and specificity for this cutoff value are 54.8% and 86.7%, respectively. (b): The AUC is 0.653 (95% CI 0.484–0.822). The cutoff value of esophageal diameter is ≥47.9 mm. The sensitivity and specificity for this cutoff value are 85.4% and 53.3%, respectively. AUC, area under the ROC curve; CI, confidence interval; ROC, receiver operating characteristic.

The ROC curve for the diagnosis of residual food in the esophagus is shown in Figure 3. The area under the ROC curve (AUC) for age was 0.702 (95% CI 0.548–0.857) (Figure 3a). The cutoff value for age was determined using the ROC curve and was found to be ≥59 years. The sensitivity and specificity of this cut‐off value were 54.8% and 86.7%, respectively. The AUC of esophageal diameter was 0.653 (95% CI 0.484–0.822) (Figure 3b). The cutoff value of the esophageal diameter was determined using the ROC curve and was found to be ≥47.9 mm. The sensitivity and specificity of this cutoff value were 85.4% and 53.3%, respectively.

### 4.4 Comparison of Patients With and Without Reflux Aspiration

Of the 27 patients who did not undergo EGD the day before POEM, reflux aspiration was identified in one patient (3.7%), and three (11.1%) showed regurgitation of the residue into the oral cavity during intubation. Of the four patients, one was in the residual food group and three were in the no‐residual food group. None of the 34 patients who underwent EGD on the day before POEM showed reflux aspiration during general anesthesia.

Table 4 summarizes the comparison between patients with and without reflux aspiration. None of the patients who had undergone reflux aspiration had undergone EGD the day before POEM, and their CRP levels on the first POD were significantly higher than those of the non‐reflux aspiration group.

**TABLE 4:** Comparison between the reflux aspiration group and the no‐aspiration group.

Characteristics	Reflux aspiration group (*n* = 4)	No‐aspiration group (*n* = 57)	*p*‐Value
Age, years (median [IQR])	53.5 (52.8–54.5)	57.0 (46.0–73.0)	0.560
Sex, Male/Female	3/1	32/25	0.629
Diagnosis			0.687
Type I achalasia, *n* (%)	3 (75.0)	43 (75.4)	
Type II achalasia, *n* (%)	1 (25.0)	9 (15.8)	
Type III achalasia, *n* (%)	0 (0.0)	2 (3.5)	
EGJOO, *n* (%)	0 (0.0)	3 (5.3)	
Duration of symptoms, years (median [IQR])	6 (4.0–7.0)	8 (3.8–15.3)	0.312
Eckardt score, points (median [IQR])	5.5 (5.0–6.3)	5 (4.0–7.0)	0.719
IRP, mmHg (median [IQR])	19.4 (14.2–24.9)	22.3 (14.6–31.7)	0.505
Dilation grade, Grade I/II/III	0/3/1	18/35/3	0.213
Type, St/Sg/aSg	0/3/1	24/23/9	0.215
Prior treatment			
Balloon dilation, *n* (%)	0 (0.0)	18 (31.6)	0.310
POEM, *n* (%)	0 (0.0)	0 (0.0)	
Surgery, *n* (%)	0 (0.0)	0 (0.0)	
Residual food at the initial EGD, *n* (%)	0 (0.0)	22 (38.6)	0.287
EGD the day before POEM, *n* (%)	0 (0.0)	34 (59.6)	< 0.05
POEM failure, *n* (%)	0 (0.0)	2 (3.5)	1
Operation time, min (median [IQR])	108 (86.5–121.8)	93 (76.8–103.5)	0.509
CRP on 1POD, mg/dL (median [IQR])	6.6 (5.6–7.1)	3.1 (1.9–5.0)	< 0.05

Grade I: d < 3.5 cm, Grade II: 3.5cm ≤ d <6.0 cm, Grade III: d ≧ 6.0 cm. St (Straight type): α ≥ 135°, Sg (Sigmoid type): 90° ≤α <135°, aSg (Advanced Sigmoid type): α < 90°. CRP, C‐reactive protein; EGD, esophagogastroduodenoscopy; EGJOO, esophagogastric junction outflow obstruction; IRP, integrated relaxation pressure; POD, post‐operative day.

## 5 Discussion

In this study, we examined the factors predicting the presence of food residues in the esophagus prior to POEM. Our results revealed that, in addition to the presence of a substantial amount of food residue during the initial EGD, patients aged <60 years and those with significant esophageal dilation observed on esophagography were more likely to have food residue before POEM.

Research on POEM and the residual food during this procedure is limited. Tanaka et al. retrospectively examined 27 cases of esophageal achalasia in which EGD was performed several hours before anesthesia induction [18]. The results showed that solid food was observed in 10 patients (37.0%). In our study, 27.6% (16/52) of patients had residual food observed the day before or on the day of POEM, which is consistent with previous reports.

Yang et al. retrospectively investigated reflux aspiration during anesthesia in 52 patients with esophageal achalasia undergoing POEM [19]. Patients followed a clear liquid diet for 48 h prior to POEM. Patients with significant food residue on their first EGD were fed a longer liquid diet. No cases of reflux aspiration were recorded during anesthesia induction; however, pneumonia was observed in two patients (3.8%, 2/52) postoperatively.

In our hospital, 14.8% of the patients who did not undergo EGD the day before POEM experienced reflux aspiration during the induction of anesthesia. In contrast, none of the patients who underwent EGD the day before POEM experienced reflux aspiration during anesthesia induction. These data suggest that EGD before POEM may prevent aspiration during the induction of general anesthesia.

In the Japanese guidelines for POEM, cleansing of the esophagus before POEM is essential to prevent contamination of the esophageal remnants into the mediastinum or thoracic/abdominal cavity [20]. However, depending on the circumstances of the facility, performing EGD in all cases on the day before POEM may prove challenging. Moreover, invasive endoscopic procedures were not necessary in several cases before POEM.

In many facilities, EGD is performed the day before POEM in cases where food residue is observed during the initial EGD. Based on the results of this study, such cases evidently required EGD the day before POEM. Additionally, younger patients or those with significant esophageal dilation were more prone to food residue accumulation.

Cleansing the esophagus the day before POEM in patients at risk for esophageal food residue may reduce the likelihood of reflux aspiration during general anesthesia induction.

This study had some limitations. First, this was a single‐center, retrospective study. Second, selection bias may have influenced group allocation. At our institution, EGD and cleansing are recommended the day before POEM to prevent reflux aspiration under general anesthesia, especially for patients with food residue on initial EGD. However, due to scheduling constraints, this was not always feasible. Although we believe this had minimal impact on outcomes, selection bias cannot be excluded. Third, most patients had type I and II achalasia, with only a few having type III achalasia and EGJOO. Fourth, the dietary habits of participants were not studied, and the reason for the high residual food content in younger patients remains unclear. Additional investigations, including those examining pre‐admission dietary habits, are warranted to further clarify this finding. In this study, patients with distal esophageal spasm (DES) and hypercontractile esophagus (HE) were excluded because of the small number of cases. However, DES and HE can lead to aspiration pneumonia without significant esophageal dilatation. Therefore, a multicenter study on these rare cases is warranted.

In conclusion, patients with residual food observed during the first EGD, younger patients, and those with significantly dilated esophageal diameters were more likely to have residual food in the esophagus. We recommend that cleansing of the esophagus be performed the day before POEM for patients at high risk of residual food.

## Conflicts of Interest

The authors declare no conflicts of interest.

## Ethics Statement

The study protocol was approved by the Ethics Committee of Hirosaki University (2023‐032).

## Consent

This study involved no invasive procedures or interventions, did not utilize any human‐derived biological samples, and was conducted solely using clinical information. In accordance with ethical guidelines, the study was implemented using an opt‐out approach.

## Data Availability

All data are presented in this paper. Further enquiries can be directed to the corresponding author. N/A
